# Some Factors Which Cause Eruption of the Teeth

**Published:** 1898-02

**Authors:** Dencer Whittles

**Affiliations:** L.D.S. (Eng.)


### Abstracts and Translations.

### SOME FACTORS WHICH CAUSE ERUPTION OF THE TEETH.

BY DENCER WHITTLES, L.D.S. (ENG.).

Many causes have been assigned to account for the eruption of teeth, and among them may be enumerated lengthening of the root, contraction of the dental sac, bone currents, and more recently the blood-pressure has been brought to bear upon the expulsion of these organs from their bony crypts.

Evolution throws much light upon the most complex structures, if we compare present highly organized creatures with the lower forms of life, which may only possess lips or stomata for the prehension of food, and of course these openings would possess some form of touch corpuscles contained therein, and so in process of time these, owing to the altered environment and change of habit, become specialized and hardened by calcification, and which epidermal appendages we call teeth.

The sources of tissues which afterwards become calcified are two,—namely, epiblast or *ectoderm*, and mesoblast or *mesoderm*. The former tissue covers the whole surface of the body and those tracts which have communication with the outside of the body.

Some may take exception to this statement that all epithelial tissues are without the body in their normal position, just because nervous matter is epiblastic in origin. I surmount that difficulty, perhaps wrongly, by viewing the whole arrangement of the central nervous system doing its utmost to get outside by sending the innumerable peripheral nerves to the surface of the body, and its ganglia being only kept in by the hard, unyielding walls of the cranium and spinal column, although its early presence has produced the many foramina for the exit of its numerous branches which are maintained until the bones turn again to earth.

Mesoblastic or connective tissue-forming element of the blastoderm gives origin to the whole of a tooth with the exception of the enamel and its skin and the nerves of the pulp (or a better term would be "dental papilla").

It is a generally accepted theory that teeth will not form with out the inflexion of the Malpighian layer of cells, although no enamel may be formed on the surface of the dentine; but the epithelial cells will be there, so that the presence of enamel on a tooth (as a foreign body) is insufficient to account for its being ejected to the free surface of the oral cavity or body. One of the earliest examples of an inflexion of epithelial tissue takes place in the human ovary, which may be described briefly as consisting of fibrous tissue, with blood-vessels, etc., and is covered by a layer of columnar epithelium. The ovules, or immature ova, are developed during fœtal life from cells which come from this germ epithelium, as it is called. They get into the interior of the ovary by a process of down-growth of this germ epithelium and subsequent isolation. As the ovule matures it sinks deeper into the ovary, but in the later stages it comes quite close to the surface, and eventually the Graafian follicle ruptures, and the mature ovum escapes from the ovary, to be conducted down the Fallopian tube to the uterus. Its ultimate destination, whether impregnated or not, is the *exterior*. The whole process bears analogy to the inflexion of the oral epithelium, with the subsequent cutting off of the tooth germ and final eruption to the surface.

The developement of the ovary shows the intimate bearing which epithelial tissue has upon connective tissue. The *first sign* of the ovary is a heaping up of epithelium at one place. Subsequently, underneath this arises the connective tissue of the ovary, and this forms the bulk of the organ, while the epithelium covers its exterior with the exception of the ovules, which have come to take a place inside the ovary by a process of ingrowing and isolation.

To make myself clear, it will be advisable to consider the mode of growth of a tooth after the cap of dentine has formed until the completion of the root, and which may be described as dental anabolism or constructive metabolism; and, secondly, the removal of those tissues around the dental germ to allow of its escape, which may be conveniently called dental follicular catabolism or destructive metabolism.

It is not necessary for me to weary you with a description of the various dentines, so let it suffice for me to draw your attention to the presence of a number of large cells (larger than seen elsewhere) containing proportionately large nuclei, at the base of the dentine papilla, from the sides of which arise the two cornua of the dental sac. These, you will observe, contain nuclei almost as big as themselves, which, taken together, suggest that they are capable of great metabolic energy. To my mind, these are the cells which propel the tooth forward until the crown is quite through into position in the case of a simple rooted tooth, or in the case of a tooth of persistent growth is being kept constantly moving forward. It is not necessary to mention those teeth whose attachments are by means of a fibrous membrane or bony ankylosis, as these two are only modifications of the simple socketed tooth, like those of man.

I believe it is generally admitted that low-formed cells, generally of the round variety, possess much activity, and to give a homely simile from the vegetable kingdom,—*e.g.*, fungi,—these are capable of displacing very heavy stones, simply by cell-division, also splitting of the bark in trees.

To return to these deep, large, papillary cells and the power of force shown by them, let me remind you what occurs if by chance opposing incisor teeth of the rodents become injured, and which condition is illustrated in the current number of the *Strand Magazine*, is not at least so infrequent as stated there. Our college specimens show where the inability to obtain food resulted in the death of the animal. If from mechanical cause, such as an overcrowded mouth, want of symmetry between teeth and jaws, a tooth is unable to erupt until the root is complete, there is much less probability of its getting into a proper position, unless the alveolus by an interstitial growth can remedy the want of propulsive force, as instanced in some cases where temporary teeth have been retained, and yet come into occlusion with the permanent teeth; and again, we all know that teeth with stunted roots do erupt, and when suspected, where a crown is afterwards required, it is well to observe the finger-nails of your patient, as I find that both appendages agree to a considerable extent in quality and quantity of growth.

In furtherance of the argument, though perhaps you will not admit is a strong one, that these large cells are pushing up the whole dentine organ, it follows that the formed dentine will also travel in the direction to which the tooth crown is to occupy; such movement may give rise to the flattening of the ends of the enamel prisms, from which we can see Tomes's processes emerging. Should not such a movement have a tendency to consolidate the enamel, for this does not commence to form until the dentine has commenced to calcify, and at this time the forward movement on the part of the mesoblastic tissues may take place in its efforts to drive out the invading ameloblasts, cells specialized from the epithelium.

I have an idea that there are some phenomena occurring during the life history of an organism, which I can only, for the want of a better term, call *physiological* irritation, as opposed to that of a *pathological* character, which would be associated with some form or other of inflammation.

I will take the physiological aspect first, which is only a means to an end: if no primary inflexion of epithelium, then no tooth will be formed; the mesoblastic tissue must be irritated to form its dentine, and subsequently the tooth will erupt, and, without enlarging upon the subject, pass on to the pathological aspect.

If from some cause or other, say prolonged irritation of the submucous tissue of the tongue from the ragged and sharp edges of a tooth, specially in the bicuspid region, the mesoblastic tissues become so lowered in vitality that they are unable to prevent the inward growth of epithelium, in other words, the balance of power for growth has been lost to the submucous tissue, and the surface epithelium has taken advantage of its neighboring tissue's infirmity and proliferates in all directions, and we have one of the varieties of cancer.

Another example, but more particularly to illustrate the force produced by multiplying cells, is the method by which dead bone, after separation by ulceration of further loss of living bone, is brought to the surface of the body, not unlike the mode by which a tooth whose enamel cap stands in a similar relation; and although enamel may not be present in sufficient quantity to be recognized, even microscopically, there must necessarily be the skin of the tooth, which we have chosen to call Nasmyth's membrane, which is now affirmed to be of epithelial origin.

Still one other example, and that is the enormous amount of bulging which occurs between the inner and outer walls of a mandible, when such is affected with myeloid sarcoma; this bulging is entirely due to an increase of growth of large cells not unlike the cells which I have called your attention to as causing the propulsion of a tooth from its osseous environment.

So, to sum up these respective cells, which may be similar owing to their power of rapid development and of propulsion,—*i.e.*, the new formation cells of granulation tissue, myeloid cells, basal dentine papillary cells, and in fact most connective-tissue cells of an anabolic character.

In the earlier part of this paper I mentioned the comparative balance of power for growth of epiblastic and mesoblastic tissues and at this point it is convenient to remind you that when the various glands, whether mucous, sweat, mammary, etc., are formed, there is always a preliminary heaping up of epithelium before invasion of the mesoblastic tissue by the aforesaid takes place, and it seems as though it was necessary to occur, to enable the Malpighian layer to obtain sufficient force to make its raid upon the bordering territory, since the power for growth in this early embryonic state is in favor of the epiblastic layer, otherwise there would not be any formation of the numerous cavities and canals which we find associated with the head and face, which are produced by the early invagination of this tissue.

Another instance of the force of power for growth is the early aberration of the enamel organ, which causes great distention of the bony structure, and was known to early surgeons as a tumor of an epithelial multilocular cystic variety. It has occurred to me whether that peculiar form of head, sometimes associated with rickets, and known as hydrocephalus, is in any way similar to an epithelial odontome, and it is due to that want of harmony which should exist between tissues, as the bone seems scarcely able to keep the cerebro-nervous system within bounds, and the consequent pressure might lead to the increase of cerebro-spinal fluid, due to the breaking down of its cells.

Assuming that the tooth in process of formation has commenced to move towards the surface of the body, whether it is a free surface or partially closed like the antrum and nasal cavity, or may be at the angle of mandible on the outside,—for it will erupt sooner or later, unless there is a stronger force to prevent it,—how is the direction determined, and how shall sufficient room be made for the passage of the crown in the case of a tooth of limited growth, or the continuous renewal of a persistently growi g tooth? In each case the first eruption is probably carried on in a similar manner.

The tissues between the forming tooth crown and the oral surface consist chiefly of the enamel organ, the inner and outer walls of the tooth-sac, bone, and muco-periosteum.

Now, most teeth before eruption have one or more points upon them, probably to assist this much-discussed process, and it is frequently observed that the lower central incisors in *genus homo* occupy less time in their eruption than the other remaining teeth of the series; the question naturally occurs, Why should this be? Is it that sufficient room having been made by a method to be described later, that the transverse diameter of the biting surface is more out of proportion with the neck when compared with other teeth. The opportunities that a child has for applying gingival massage to the mandible by making it a lever of considerable power, induced by nerve-irritation, such irritation being only a means to an end, and to produce atrophy of these tissues between the almost completed crown and the surface of the oral cavity.

We have all observed in our experience that the cells forming the wall of the dental sac can either build up or pull down the structures they are destined to be associated with, as instanced in exostosis and absorption of roots of teeth.

While the enamel organ is in contact with the dental sac, after a certain amount has been calcified, absorption begins to take place in the neighborhood of the forming crown, and from my observation I have noticed that when absorption is going on the blood-vessels in the immediate vicinity are much more dilated than when a deposition of tissue is taking place elsewhere, and in the case for a passage being made for the erupting tooth, such dilated vessels anastomose with those supplying the stellate reticulum, which I believe to be due to this being a ready market for the lime salts in the enamel organ only just taken from the bone by those cells having taken on an absorption function.

Supposing, now, that the bone has been removed by these osteo- or cementoblastic cells, there is only the muco-periosteum to be removed; this is probably subjected to atrophy on account of the pressure cutting off the nourishment from below, and the separation of those two epithelial layers of cells, now atrophied, open sufficiently wide to allow the tooth to escape, and the portion of free epithelial tissue with a little mesoblastic material from the extreme tips of the dental sac now form the little elevations or dental cushions seen in and around the teeth.

The atrophied neck of the enamel organ, termed the gubernaculum, has kept back the mesoblastic tissues, and if we examine young, dry mandibles it is easy to find a number of holes behind the temporary teeth; this band of tissue was supposed by the older anatomists to pull up the tooth when ready; it may serve as an aid to the process by keeping patent a small orifice for the evacuation of the tooth, and it has occurred to me whether the obliteration of this gubernaculum is the direct cause of that variety of odontoma we commonly describe as dentigerous cyst.

The supposed contraction of the tooth-sac has been brought to bear by some writers as a method of eruption; if this is so, it is difficult to account for the dilated sacs not infrequently found calcified in follicular odontomes. Where teeth of persistent growth occur, especially those whose position is maintained by their curved shape, the contraction of the dental sac would serve little purpose, as it is found that the older the animal grows, the greater the calibre of the still growing tooth, so that there is always some osseous tissue to be absorbed before they can be pushed outward; these are well illustrated by the incisors of the elephant and rodents, and canines of many others.

In my opinion the only way that the tooth-sac can assist the first passage out of any tooth is very much like the driving of a wedge, just as the fœtal head dilates the os uteri previous to its expulsion.—*Journal British Dental Association.*

